# Royal Jelly Modulates Oxidative Stress and Apoptosis in Liver and Kidneys of Rats Treated with Cisplatin

**DOI:** 10.1155/2011/981793

**Published:** 2011-08-01

**Authors:** Ali Karadeniz, Nejdet Simsek, Emre Karakus, Serap Yildirim, Adem Kara, Ismail Can, Fikrullah Kisa, Habib Emre, Mehmet Turkeli

**Affiliations:** ^1^Department of Physiology, Faculty of Veterinary Medicine, University of Atatürk, 25240 Erzurum, Turkey; ^2^Department of Histology and Embryology, Faculty of Veterinary Medicine, University of Atatürk, 25240 Erzurum, Turkey; ^3^Department of Pharmacology and Toxicology, Faculty of Veterinary Medicine, University of Atatürk, 25240 Erzurum, Turkey; ^4^Department of Physiology, Faculty of Medicine, University of Atatürk, 25240 Erzurum, Turkey; ^5^Department of Nephrology, Faculty of Medicine, University of Yüzüncü Yıl, 65080 Van, Turkey; ^6^Department of Medical Oncology, Faculty of Medicine, University of Atatürk, 25240 Erzurum, Turkey

## Abstract

Cisplatin (CDDP) is one of the most active cytotoxic agents in the treatment of cancer and has adverse side effects such as nephrotoxicity and hepatotoxicity. The present study was designed to determine the effects of royal jelly (RJ) against oxidative stress caused by CDDP injury of the kidneys and liver, by measuring tissue biochemical and antioxidant parameters and investigating apoptosis immunohistochemically. Twenty-four Sprague Dawley rats were divided into four groups, group C: control group received 0.9% saline; group CDDP: injected i.p. with cisplatin (CDDP, 7 mg kg^−1^ body weight i.p., single dose); group RJ: treated for 15 consecutive days by gavage with RJ (300 mg/kg/day); group RJ + CDDP: treated by gavage with RJ 15 days following a single injection of CDDP. Malondialdehyde (MDA) and glutathione (GSH) levels, glutathione S-transferase (GST), glutathione peroxidase (GSH-Px), and superoxide dismutase (SOD) activities were determined in liver and kidney homogenates, and the liver and kidney were also histologically examined. RJ elicited a significant protective effect towards liver and kidney by decreasing the level of lipid peroxidation (MDA), elevating the level of GSH, and increasing the activities of GST, GSH-Px, and SOD. In the immunohistochemical examinations were observed significantly enhanced apoptotic cell numbers and degenerative changes by cisplatin, but these histological changes were lower in the liver and kidney tissues of RJ + CDDP group. Besides, treatment with RJ lead to an increase in antiapoptotic activity hepatocytes and tubular epithelium. In conclusion, RJ may be used in combination with cisplatin in chemotherapy to improve cisplatin-induced oxidative stress parameters and apoptotic activity.

## 1. Introduction

*Cis-Diaminedichloroplatinum* (II) (CDDP), commonly known as cisplatin, has been established as a potent chemotherapeutic agent administered to treat a variety of cancers such as ovarian, bladder, testicular, head and neck, and uterine cervix carcinomas [1, 2]. The major dose-limiting side effect of cisplatin is its nephrotoxicity and hepatotoxicity, and nephrotoxicity can result in severe nephropathy leading to acute renal failure. In several studies, it had been documented that injection of cisplatin produced a marked decrease in renal blood flow and glomerular filtration rate. The alterations in the kidney and liver functions induced by cisplatin are closely associated with an increase in lipid peroxidation and reactive oxygen species (ROS) in the tissues [3, 4]. In addition, cisplatin may have some mechanisms of liver injury such as functional and structural mitochondrial damage, apoptosis, and perturbation in Ca^2+^ homeostasis [5, 6].

Reactive oxygen species (ROS) such as hydrogen peroxide, hydroxyl radical, singlet oxygen, superoxide anion, and peroxyl radical are formed inside cells by exposure to several endogenous and exogenous agents, causing damage to many important biomolecules that have been implicated in several diseases [7]. These prooxidants are kept in check by endogenous antioxidants, but under disease conditions, the balance is shifted in favor of prooxidants, leading to oxidative stress. Excess ROS causes significant oxidative damage by attacking biomolecules such as membrane lipids, DNA, and proteins in cells [8]. The oxidative stress is associated with many disease states including neurological diseases such as Alzheimer's brains and Parkinson's disease, chronic heart disease, and kidney and liver diseases [9]. Endogenous antioxidants such as reduced glutathione (GSH), glutathione peroxidase (GSH-PX), superoxide dismutase (SOD), catalase (CAT) are compounds that act as free radical scavengers. These antioxidants are electron donors and react with the free radicals to form harmless products such as water. Therefore, antioxidants protect against oxidative stress and prevent damage to cells [10].

Some natural products, such as lycopen [11], grape seed extract [12], green tea [13], and caffeic acid phenethyl ester [14], are demonstrated to have a protective role against cisplatin-induced kidney and liver oxidative damages. Hence, there has been considerable interest in role of naturally originated agents for the treatment of kidney and liver diseases. Recently, royal jelly (RJ) has received particular attention because of studies that have reported that it is a highly efficient antioxidant and has free radical scavenging capacity [4, 15]. Royal jelly is a secretion produced by the hypopharyngeal and mandibular glands of worker honeybees (*Apis mellifera*). It contains many important compounds with biological activity such as free amino acids, proteins, sugars, fatty acids, minerals, and vitamins [16]. So far, RJ has been demonstrated to possess several physiological activities in experimental animals, including vasodilative and hypotensive activities [17], the induction of decrease in serum cholesterol levels [18], antimicrobial [19], antiallergic [20], anti-inflammatory [21], immunomodulatory [22, 23], and antioxidant properties [16]. In addition, Kanbur et al. [24] revealed the protective effect of RJ against paracetamol-induced liver damage in mice. 

Apoptosis is a gene-regulated event related to special morphological changes such as shrinkage of cell, chromatin condensation, and DNA damages [25]. A number of factors contributed to apoptotic mechanism, that are believed to play a key role in two main families of proteins including cysteine proteases called caspase enzymes (especially caspase 3, 8, and 9) and Bcl-2 family. Caspase 3, 8, and 9 enzymes play an effective role in apoptotic process of the liver and the kidney. Caspase-3, the most important member of caspase family, is responsible for many biochemical mechanisms of apoptosis that lead to the cleavage of nuclear and cytosolic substrates, chromatin condensation, fragmentation of DNA, and apoptotic bodies [26, 27]. Whereas, Bcl-2, which is acts either antiapoptotic (Bcl-*x*_*L*_) or proapoptotic (Bcl-*x*_*S*_), Bcl-*x*_*L*_, an important member of this family, prevents apoptotic activity by blocking cytochrome *c *release from mitochondria [28].

Thus, the aim of the present study was to investigate the protective effect of royal jelly in cisplatin-induced oxidative damages and apoptotic changes of rat liver and kidney by biochemical and immunohistochemical methods.

## 2. Results

### 2.1. Biochemical Findings

No animal death was observed in the control and other RJ- and CDDP-treated groups. The effects of RJ and CDDP on serum biochemical parameters are presented in Table 1. CDDP-treated rats showed a significant increase in ALT, AST activities, and creatinine levels (*P* < 0.05). However, pretreatment of rats with RJ significantly inhibited these CDDP-induced elevations of ALT and AST activities and creatinine levels. In addition, RJ alone induced changes are not significant for all biochemical parameters tested.

The changes in MDA and GSH levels and GSH-Px, GST, and SOD activities in the liver and kidneys are shown in Table 2. The MDA levels in the liver and kidneys of animals that were administered CDDP alone were observed to display significant increase compared with control group, and this increase was found to be statistically significant (*P* < 0.05). This increase was attenuated by pretreatment with RJ. Also, significantly (*P* < 0.05) reduced levels of GSH and GSH-Px, GST, and SOD activities were seen in the liver and kidney tissues of CDDP-treated rats compared with the control groups. Pretreatment of rats with RJ alleviated these CDDP-induced decreases (Table 2). Alone RJ treatment resulted in a significant increase in GSH levels and GSH-Px, GST, and SOD activities accompanied with a significant decrease in MDA compared to the control values.

### 2.2. Histochemical and Immunohistochemical Findings

The livers and kidneys of the control and the RJ-only groups showed normal histological structures. In the CCDP-treated groups, significant histological alterations consisting of congestion, dilatation, epithelial vacuolization, and infiltration of mononuclear cells in both liver and kidney tissues took place. On the other hand, these changes were decreased in the RJ + CCDP treated animals.

**Figure 1:**
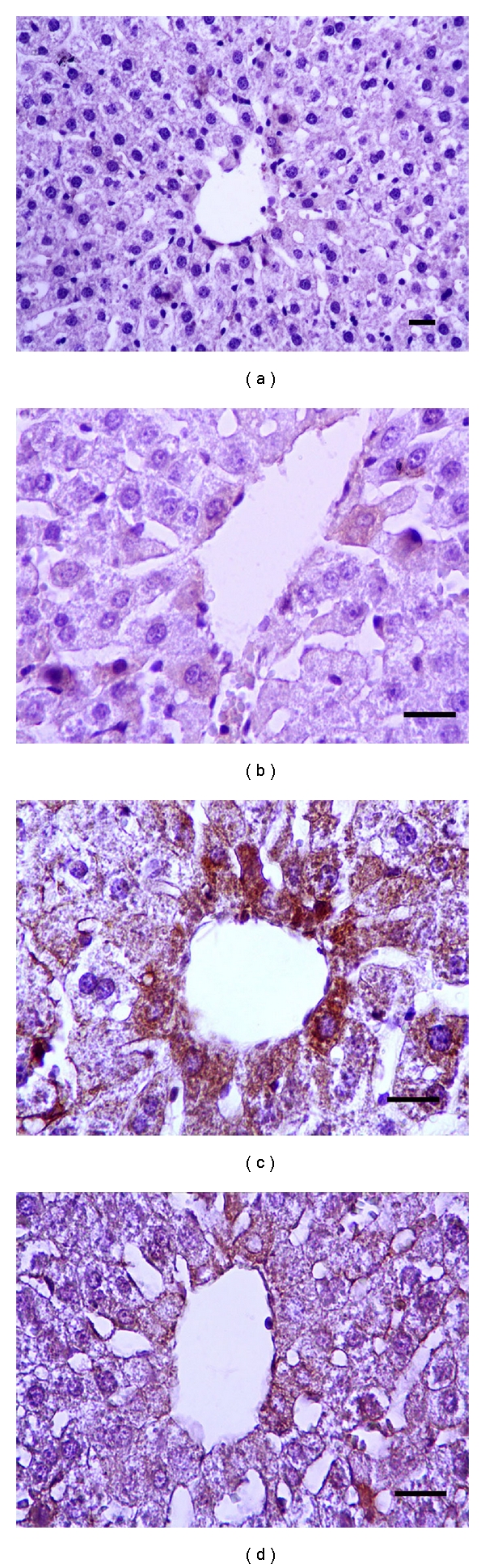
Caspase-3 positive reactions in rat livers. (a) Control, (b) RJ, (c) CDDP, (d) RJ + CDDP (streptavidin-biotin peroxidase staining), bar 20 *μ*m.

**Figure 2:**
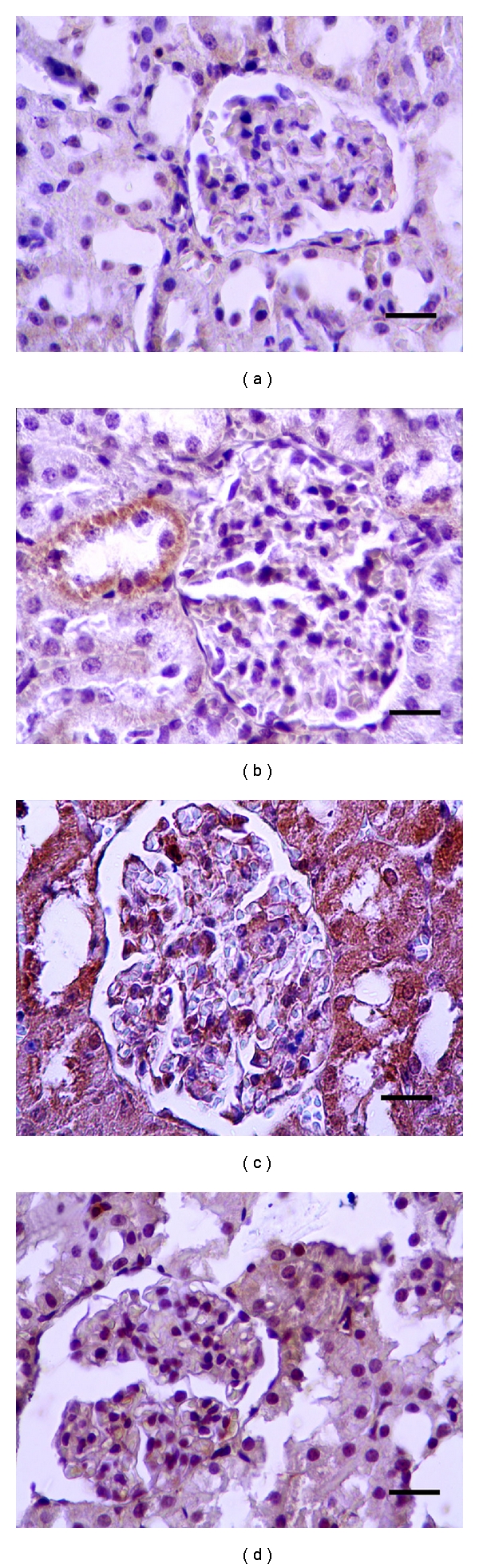
Caspase-3 positive reactions in rat kidneys. (a) Control, (b) RJ, (c) CDDP, (d) RJ + CDDP (streptavidin-biotin peroxidase staining), bar 20 *μ*m.

**Figure 3:**
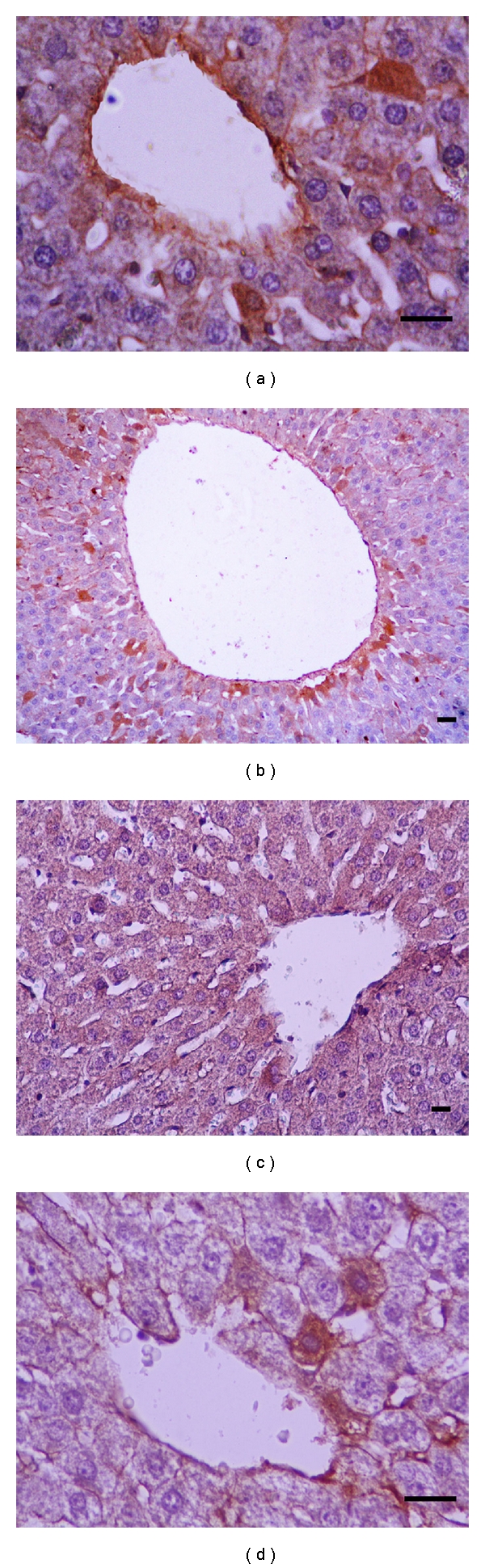
Bcl-*x*_*L*_ positive reactions in rat livers. (a) Control, (b) RJ, (c) CDDP, (d) RJ + CDDP (streptavidin-biotin peroxidase staining), bar 20 *μ*m.

**Figure 4:**
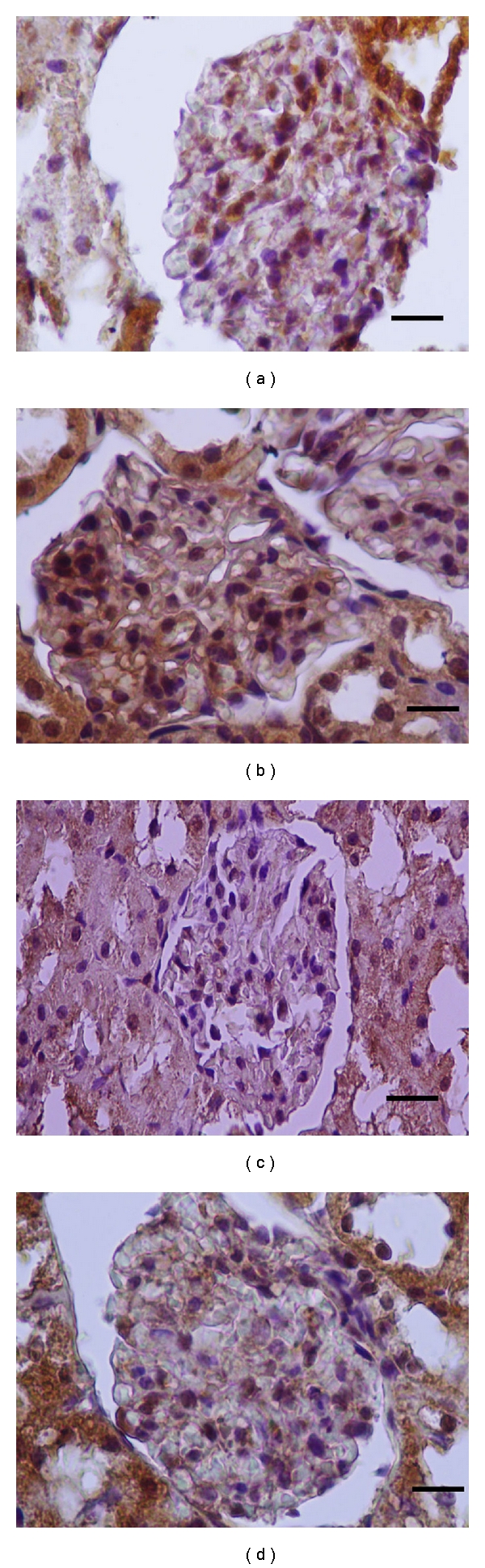
Bcl-*x*_*L*_ positive reactions in rat kidneys. (a) Control, (b) RJ, (c) CDDP, (d) RJ + CDDP (streptavidin-biotin peroxidase staining), bar 20 *μ*m.

In this study, apoptotic and antiapoptotic immunepositive reactions in the liver and kidney were investigated with caspase-3 and Bcl-*x*_*L*_ antibodies, respectively. Comparison of apoptotic and antiapoptotic activities groups are shown in Figures 1, 2, 3, and 4. Caspase-3 immune reactive cells were intensely observed around the central vein in the liver (Figure 1) and proximal tubule epithelium and glomerulus in kidney of CDDP group (Figure 2). We observed that the number of apoptotic cells were increased in the liver and kidneys of CDDP group when compared with other groups, but, in the RJ + CDDP group, there were significantly decreased apoptotic cell numbers (*P* < 0.05, Table 3). In addition, RJ treatment interestingly prevented degenerative changes and decreased the caspase-3 activity in proximal tubules according to CDDP groups (Figure 2). Bcl-*x*_*L*_ immune reactive concentration was observed around central vein in liver (Figure 3) and in cortical tubules, capsula glomeruli, and podocytes of kidney of the other groups when compared to CCDP group (Figure 4). On the other hand, Bcl-*x*_*L*_ positive reactions were increased in RJ group compared to control group (Figures 3 and 4).

## 3. Discussion

Recent studies suggest that using plant-derived chemopreventive agents in combination with chemotherapy can enhance the efficacy of chemotherapeutic agents and lower their toxicity to normal tissues [4, 17, 24, 29–31]. This study investigates the effects of RJ on CDDP-induced liver and kidney damages. Animals that received CDDP showed severe biochemical and histological changes in serum and tissues. On the other hand, pretreatment with RJ caused a significant improvement in tested parameters, which were significantly altered by CDDP administration. Serum creatinine concentration is commonly used as a measure of renal function in clinical practice [32]. In our study, Table 1 shows the increase in serum creatinine concentrations. In the present study, the creatinine concentration of the animals that were treated by CDDP alone was observed to have increased. Also accompanying histological findings show severe degeneration of cortical tubular cells in the kidney of CDDP-treated rats. Some authors have also described the increasing effects of CDDP on creatinine levels and histological changes [33–35]. In this study, the administration of RJ before CDDP treatment significantly prevented the increase in serum creatinine level compared to the CDDP alone treated group. Moreover, histological findings showed that RJ administration caused less degenerative alterations in the liver and kidneys as in previous studies.

The increase in AST and ALT activities in the CDDP-alone group was found to be related to damage in the liver and change in hepatic functions. The rise in levels of serum AST and ALT has been attributed to the damaged structural integrity of the liver, because these are normally located in the cytoplasm and are released into the circulation after hepatic damage [36]. Greggi Antunes et al. [33], Parlakpinar et al. [34], and Mora et al. [35] have also described the increasing effects of CDDP on changes in serum AST and ALT activities. On the other hand, pretreatment with the RJ remarkably inhibited CDDP-induced liver damage as evidenced by decreased serum activities of AST and ALT (Table 1). Additionally, histopathological analysis of liver sections indicated a moderate centrilobular necrosis and lymphocytic infiltration in rats treated with RJ+CDDP compared with CDDP alone.

Cisplatin acts on cancer cells by releasing free radicals such as superoxide radicals, hydroxy radicals, peroxyl radicals, and singlet oxygen, which at the same time damage liver and kidney cells. Free radicals are known to attack the highly unsaturated fatty acids of the cell membrane to induce lipid peroxidation, which is considered a key process in many pathological events and is one of the reactions induced by oxidative stress [1, 37]. Cisplatin treatment causes an increase in lipid peroxide levels and a decrease in the activities of antioxidant enzymes that protect against lipid peroxidation in the tissues such as liver and kidney [31, 38]. Many cellular pathways have been suggested to contribute to induction of a state of oxidative stress and lipid peroxides. For example, it is possible that cisplatin-induced oxidative stress and cytochrome P450 2E1-(CYP2E1-) mediated oxidative stress synergize to produce hepatotoxicity [39]. On the other hand, Safirstein [40] stated that the main targets of cisplatin in kidney are proximal straight and distal convoluted tubules, where it accumulates and encourages cellular damage by multiple mechanisms such as oxidative stress, DNA damage, and apoptosis.

The present study indicated that lipid peroxidation (MDA) in liver and kidneys significantly increased in rats treated with cisplatin alone. This result agrees with previous studies which have demonstrated the involvement of oxidative stress and lipid peroxidation in CDDP-induced liver and kidney toxicities [4, 11, 13, 14, 31]. Prophylactic RJ treatment significantly ameliorated the increase in liver and kidney MDA levels. The RJ contains biologically active amino acids such as aspartic acid, cysteine, cystine, tyrosine, glycine, lysine, leucine, valine, and isoleucine. As indicated by previous researchers, the antioxidant effect of RJ may be related to its free amino acid content [41].

Reactive oxygen species (ROS) such as hydrogen peroxide, superoxide anions, and hydroxyl radicals are generated under normal cellular conditions and are immediately detoxified by major scavenger enzymes (glutathione based enzymes such as GSH-Px, GST, SOD, and CAT. However, excessive ROS production by CDDP causes antioxidant imbalance and leads to lipid peroxidation and antioxidant depletion [42]. In our study, the major scavenger enzymes activities (GSH, GSH-Px, GST, and SOD) were significantly decreased in liver and kidneys of cisplatin-treated rats. This result may be connected with the CDDP-induced increase in free radical generation or a decrease in amounts of protecting enzymes against lipid peroxidation. In previous studies, CDDP has been found to have a peroxidative effect on the liver and kidney tissues [4, 14, 34]. However, the treatment with RJ ameliorated the cisplatin-induced liver and kidney damages due to free radical production. Meanwhile, the elevated GSH level, activities of GSH-Px, GST, and SOD enzymes in the CDDP plus RJ group implied a decrease in the number of free radicals after cisplatin administration and reflected that these enzymes played important roles in scavenging of free radical. Our findings are similar to results of other investigators such as Al-Majed et al. [43], Aleisa et al. [44], and Arafa [30] for kidney and liver tissues in which cisplatin and carboplatin injections caused low GSH, GSH-Px, SOD, and nitric oxide levels. Furthermore, in this study, it was observed that levels of GSH, activities of GST, GSH-Px, and SOD in CDDP plus RJ-treated group were higher than in the CDDP group. These results suggested that RJ has a supporting effect on the antioxidant system because of increases in GSH, GSH-Px, GST, and SOD activities. Recently, it has been demonstrated that RJ prevents carbon tetrachloride- [15], cadmium- [45], and paracetamol- [24] induced liver toxicity genotoxicity and nephrotoxicity, respectively.

A number of studies reported that CDDP has been found to have an apoptotic effect on kidney and liver [28, 46–48]. Apoptosis is characterized by phosphatidylserine externalization, membrane budding, cell shrinkage, and chromatin condensation. The apoptotic caspase enzymes are named as initiator caspases (e.g., 2, 8, 9, 10 and 12) and effector caspases (e.g., 3, 6, and 7). The effector caspases act via the activation of initiator caspases which trigger the apoptotic process [26, 27, 49], whereas Bcl-2 family proteins, which are generally acts antiapoptotic, are inhibited cytochrome c release from mitochondria and programmed cell death-inducing enzymes [28, 50, 51]. Bcl-*x*_*L*_ is a member of the Bcl-2 family, which has been observed to regulate apoptosis in response to chemotherapy [51, 52]. Bcl-*x*_*L*_ may be inhibiting apoptotic process by forming an inhibitory complex with procaspase-9 and Apaf-1, whose overexpressions are delay or inhibit apoptosis [53], as well as provide a true survival advantage in cell treated with chemotherapeutic agents [51]. In this study, an increased immune positive staining of Bcl-*x*_*L*_ were observed in both liver and kidney sections in RJ + CCDP compared to CCDP group. These findings suggested that the overexpression pattern of the Bcl-*x*_*L*_ family protein caused by royal jelly might be required in chemotherapy. 

Caspase-3 has been identified as being a key mediator of apoptosis in mammalian cells. Apoptosis is caused by activation of the initiator caspases-8 and caspase-9, which trigger the effector caspase-3 [54]. Renal cell death is a consequence of cisplatin treatment during chemotherapy and is one of the major factors limiting its use [47, 55]. In the proximal tubules of cisplatin-treated kidneys may be showed histological changes such as apoptosis and oncosis [49]. This is similar to results with cisplatin in vitro where apoptosis or oncosis is dependent on the concentration and length of exposure [56]. In vitro, caspase-3 is activated during cisplatin-induced renal cell apoptosis [56], but the mechanisms responsible for caspase-3 activation are not fully understood. In this study, an increased caspase-3 expression was observed liver and kidneys of rats treated with CCDP compared to control group. This results of this study were in agreement with those of the previous reports [29, 48, 57]. The cisplatin-induced apoptosis is believed to be the result of DNA damage [46, 47]. These results demonstrate that the caspase-3 activity increase might be correlated with CCDP-induced apoptosis. Besides, RJ pretreatment before CCDP injection in both liver and kidney lowered apoptotic immune staining cell numbers but increased antiapoptotic Bcl-*x*_*L*_ activity. Therefore, cisplatin-induced apoptosis, at least in part, may be prevented by royal jelly. 

## 4. Conclusion

In conclusion, CDDP treatment induces liver and kidney injury as indicated by the elevation of serum biochemical parameters, the decline of the antioxidant activity, and the increases of caspase-3 immune staining cells number. The excessive production of free radicals is one of the main reasons for the changes above. However, pretreatment with RJ was found to reduce the CDDP-induced liver and kidney chemical changes and apoptotic cell numbers. This protection may be due to antiapoptotic, antioxidant, and free radical scavenging activity of royal jelly and its components. Therefore, RJ may be of help to prevent liver and kidney toxicity manifested by CDDP chemotherapy.

## 5. Material and Methods

### 5.1. Materials

Royal jelly was obtained from Arı Mühendislik Company, Ankara, Turkey, and was stored at −20°C until used. Doses of 300 mg/kg RJ were dissolved in distilled water and given orally for 10 consecutive days to groups of RJ and RJ + CDDP rats. The injectable form of CDDP (Ebewe and Liba, Istanbul, Turkey) was purchased from local pharmacies. All other chemicals were obtained from Sigma (St. Louis, Mo, USA).

### 5.2. Animals and Treatments

The Atatürk University's Experimental Research Centre, Erzurum, Turkey, provided adult female Sprague Dawley rats, 180 ± 20 g, and 6–8 weeks old. The animals were kept in metal cages at a temperature of 22–24°C and a 12-hour light/dark cycle during the study and were fed with standard commercial rat food and tap water. All experiments in this study were approved by the Local Ethics Board of Animal Experiments in Atatürk University. The animals were divided into 4 groups, each with 6 rats, according to their experimental treatment. Control group rats (C) received 0.9% saline. Royal jelly group rats (RJ) were treated for 15 consecutive days by gavage with RJ (300 mg/kg/day). Cisplatin group rats (CDDP) received a single-dose injection of CDDP (7 mg/kg body weight i.p.; Ebewe and Liba, Istanbul, Turkey) all at once. Royal jelly plus cisplatin group rats (RJ + CDDP) received single oral doses of royal jelly (300 mg/kg/day p.o.) for 15 consecutive days following a single-dose i.p. injection of CDDP (7 mg/kg^−1^).

### 5.3. Sample Collection and Biochemical Assays

Twenty-four hours after the final RJ and CDDP treatments, all animals were anaesthetized with an i.p. injection of 60 mg sodium pentobarbitone per kg body weight and then sacrificed by cervical dislocation. Blood samples were collected into tubes and centrifuged at 3000 g for 10 min. Sera were separated and then stored at −80°C until analysed. The liver and kidneys were removed, washed with physiological saline solution, and stored at −80°C until analysis. All tissues were maintained at +4°C throughout preparation. A portion of liver and kidneys (1 : 9, w/v) for all assays were homogenized in a 0.9% NaCl solution with an OMNI TH international homogenizer (Warrenton, Va, USA). Tissue homogenates were centrifuged for 15 minutes at 15.000 g, and then the clear supernatants were removed for analyses. For the measurement of malondialdehyde (MDA) levels, the method described by Ohkawa et al. [58] was used. Measurements of tissue glutathione (GSH), glutathione-peroxidase (GSH-Px), glutathione-S-transferase (GST), and superoxide dismutase (SOD) activities were performed in accordance with the methods described by Tietze [59] and Anderson [60], Paglia and Valentine [61], Habig et al. [62], and Sun et al. [63], respectively. The results were expressed as nmol/g protein for MDA, *μ*mol/g protein for GSH and GST, and U/g protein for GSH-Px and SOD. Alanine aminotransferase (ALT) and aspartate aminotransferase (AST) activities and creatinine concentration were measured by using auto analyser (AU-2700; Olympus, Tokyo, Japan).

### 5.4. Histochemical and Immunohistochemical Examination

The liver and kidney samples were fixed in 10% buffered neutral formalin and embedded in paraffin. The paraffin blocks were cut 5–7 *μ*m thick and stained with Mallory's triple stain modified by Crossman. Stained specimens were examined under a light microscope. Apoptotic cells were determined by immunohistochemical method (streptavidin-biotin-peroxidase staining) seen as brown colour. For immunohistochemical examination, primary antibodies monoclonal caspase-3 (dilution: 1/25, Biovision-3015-100) and Bcl-*x*_*L*_ (dilution: 1/50 Santa cruz-sc-8392) and biotinylated secondary antibody (DAKO-Universal LSAB Kit-K0690) were used. The binding sites of antibody were visualized with DAB (Sigma) and evaluated by high-power light microscopic examination (*Nikon i50*). All apoptotic and antiapoptotic staining cells were estimated with an image processing system (Kameram SLR, 1.6.1.0, Mikro Sistem Ltd. Şti., Turkey). For each specimen, caspase-3 and Bcl-*x*_*L*_ immunreactivities were examined in 10 randomly selected areas of approximately X20 objective. 

The microscopic scoring of sections was carried out by a histopathology laboratory technician and histologist. This scale has composed to *A*: weak in ≤25% of tissue; *B*: mild in ≤50% of tissue; *C*: moderate in ≤75% of tissue; *D*: very strong in ≥75% of tissue. The average degeneration intensity was calculated as ((*A* × 1) + (*B* × 2) + (*C* × 3) + (*D* × 4))/(*A* + *B* + *C* + *D*) and reported as follows: + = 0.00–1.00; ++  =  1.01–2.00; +++  =  2.01–3.00; ++++  =  3.01–4.00. The scores were derived semiquantitatively using light microscopy on the preparations from each animal and were reported as follows: none: −, mild: +, moderate: ++, severe: +++, and very strong: ++++.

### 5.5. Statistical Analysis

For statistical analysis, differences between the groups were tested by the analysis of variance (ANOVA) followed by Duncan's post hoc test using SPSS 17.0 for Windows XP (SPSS Inc., Chicago, Ill). A value *P* < 0.05 was considered significant. All data were expressed as mean averages, ± S.E.M.

## Figures and Tables

**Table 1:** Effects of royal jelly on selected biochemical parameters in rats treated with CDDP.

Parameters	Groups			
	C	CDDP	RJ	RJ + CDDP

ALT (IU/L)	27.45 ± 1.40^a^	80.50 ± 2.50^b^	29.50 ± 1.70^a^	35.85 ± 2.25^c^
AST (IU/L)	55.8 ± 1.55^a^	90.80 ± 3.50^b^	58.8 ± 5.30^a^	65.75 ± 2.50^c^
Creatinine (mg/dL)	1.05 ± 0.60^a^	3.15 ± 0.50^b^	1.02 ± 0.51^a^	2.15 ± 0.55^c^

Different superscripts a, b, c in the same row indicate significant differences between groups (*n* = 6).

*P* < 0.05, means ± S.E.M. C: Control, RJ: royal jelly, CDDP: cisplatin.

**Table 2:** The effects of royal jelly administration on MDA, GSH, GSH-PX, GST, and SOD levels in liver and kidneys of rats treated with CDDP.

Groups	Parameters									
	MDA		GSH		GSH-Px		GST		SOD	
	(nmol/g protein)		(*μ*mol/g protein)		(U/g protein)		(*μ*mol/g protein)		(U/g protein)	
	Liver	Kidney	Liver	Kidney	Liver	Kidney	Liver	Kidney	Liver	Kidney

C	38.50 ± 9.50^a^	13.50± 1.40^a^	130.75 ± 9.50^a^	40.75± 0.25^a^	95.50 ± 8.40^a^	95.50 ± 6.50^a^	75.50± 6.50^a^	45.50 ± 12.50^a^	680.50 ± 47.30^a^	630.50 ± 22.70^a^
RJ	37.50 ± 6.90^a^	16.45± 0.75^a^	133.30 ± 7.50^a^	41.30± 0.30^a^	109.50 ± 6.50^a^	90.50 ± 10.30^a^	92.80 ± 6.30^a^	42.50 ± 10.75^a^	755.50 ± 43.80^a^	620.75 ± 35.50^a^
CD DP	60.50 ± 6.50^b^	32.50 ± 2.40^b^	92.50 ± 8.50^b^	21.50 ± 0.20^b^	70.50 ± 7.30^b^	42.75 ± 8.20^b^	53.70 ± 7.50^b^	18.50 ± 9.50^b^	510.20 ± 45.50^b^	480.30 ± 43.30^b^
RJ + CDDP	49.50 ± 6.50^c^	21.30 ± 0.90^c^	115.80 ± 7.90^c^	37.50 ± 0.20^c^	90.80 ± 6.50^c^	72.50 ± 9.50^c^	63.50 ± 7.70^c^	30.50 ± 13.50^c^	595.50 ± 28.50^c^	520.30 ± 24.50^c^

Different superscripts a, b, c in the same column indicate significant differences between groups (*n* = 6).  *P* < 0.05, means ± S.E.M. C: Control, RJ: royal jelly, CDDP: cisplatin.

**Table 3:** Semiquantitative analysis of caspase-3 and Bcl-*x*_*L*_ reactivity in liver and kidney.

Groups	Immunstaining density			
	Caspase-3		Bcl-*x*_*L*_	
	Liver	Kidney	Liver	Kidney

C	+	++	++++	++++
RJ	+	+	++++	++++
CCDP	++++	+++	++	+
RJ + CCDP	++	++	+++	++

Caspase-3 and Bcl-*x*_*L*_ reaction density was estimated as follows: none: 0, weak: +, moderate: ++, strong: +++, very strong: ++++. C: Control, RJ: royal jelly, CDDP: Cisplatin.
